# Associations between Heart Rate Variability Parameters and Housing- and Individual-Related Variables in Dairy Cows Using Canonical Correspondence Analysis

**DOI:** 10.1371/journal.pone.0145313

**Published:** 2015-12-21

**Authors:** Levente Kovács, Fruzsina Luca Kézér, Mikolt Bakony, Levente Hufnágel, János Tőzsér, Viktor Jurkovich

**Affiliations:** 1 MTA–SZIE Large Animal Clinical Research Group, Üllő-Dóra major, H-2225, Hungary; 2 Institute of Animal Husbandry, Faculty of Agricultural and Environmental Science, Szent István University, Páter Károly u. 1, Gödöllő, H-2100, Hungary; 3 Rumino-Vet Bt., Csillés utca 2., Érd, H-2030, Hungary; 4 Institute of Crop Production, Faculty of Agricultural and Environmental Science, Szent István University, Páter Károly u. 1, Gödöllő, H-2100, Hungary; 5 Department of Animal Hygiene, Herd Health and Veterinary Ethology, Faculty of Veterinary Science, Szent István University, István u. 2, Budapest, H-1078, Hungary; University of Florida, UNITED STATES

## Abstract

We investigated the associations between heart rate variability (HRV) parameters and some housing- and individual-related variables using the canonical correspondence analysis (CCOA) method in lactating Holstein-Friesian dairy cows. We collected a total of 5200 5-min interbeat interval (IBI) samples from 260 animals on five commercial dairy farms [smaller-scale farms with 70 (Farm 1, n = 50) and 80 cows per farm (Farm 2, n = 40), and larger-scale farms with 850 (Farm 3, n = 66), 1900 (Farm 4, n = 60) and 1200 (Farm 5, n = 45) cows. Dependent variables included HRV parameters, which reflect the activity of the autonomic nervous system: heart rate (HR), the root mean square of successive differences (RMSSD) in IBIs, the standard deviation 1 (SD1), the high frequency (HF) component of HRV and the ratio between the low frequency (LF) and the HF parameter (LF/HF). Explanatory variables were group size, space allowance, milking frequency, parity, daily milk yield, body condition score, locomotion score, farm, season and physical activity (lying, lying and rumination, standing, standing and rumination and feeding). Physical activity involved in standing, feeding and in rumination was associated with HRV parameters, indicating a decreasing sympathetic and an increasing vagal tone in the following order: feeding, standing, standing and rumination, lying and rumination, lying. Objects representing summer positioned close to HR and LF and far from SD1, RMSSD and HF indicate a higher sympathetic and a lower vagal activity. Objects representing autumn, spring and winter associated with increasing vagal activity, in this order. Time-domain measures of HRV were associated with most of the housing- and individual-related explanatory variables. Higher HR and lower RMSSD and SD1 were associated with higher group size, milking frequency, parity and milk yield, and low space allowance. Higher parity and milk yield were associated with higher sympathetic activity as well (higher LF/HF), while individuals with lower locomotion scores (lower degree of lameness) were characterized with a higher sympathetic and a lower vagal tone (higher HR and LF/HF and lower RMSSD and SD1). Our findings indicate that the CCOA method is useful in demonstrating associations between HRV and selected explanatory variables. We consider physical activity, space allowance, group size, milking frequency, parity, daily milk yield, locomotion score and season to be the most important variables in further HRV studies on dairy cows.

## Introduction

Stress affects many physiological systems including the cardiovascular system, which is controlled by the autonomic nervous system [1]. Heart rate variability (HRV), i.e. the short-term fluctuations in the length of successive cardiac interbeat intervals (IBI) is essentially based on the antagonistic oscillatory influences of the sympathetic and parasympathetic nervous system on the nodus sinuatrialis of the heart [2]. It thus reflects the prevailing balance of the sympathetic and the parasympathetic (vagal) tone. As stress responses are accompanied by the rapid increase in sympathetic activity and a parallel decrease in vagal activity [3], HRV has been increasingly used in farm animals, such as cattle, swine, sheep and poultry as an indicator for the response of the autonomic nervous system (ANS) to stress [4]. According to our recent review on the welfare implications of HRV in dairy cattle [5] several HRV indices are useful in investigating stress caused by technological elements or diseases.

So far, cattle HRV studies have been carried out mainly on small-scale experimental farms and involved generally 6–10 cows per group. These studies focused on short-term physiological and behavioral responses of animals in relation with breed or different milking systems [6], pain [7], or the effects of human proximity [8] in well controlled situations. According to our knowledge, there is only one study describing the effects of body posture and physical activity on HRV in dairy cows [9] that was also performed in a controlled test situation on a small-scale experimental farm. Welfare concerns of high-yielding cows necessitate field studies on the evaluation of stress by analyzing HRV on commercial farms. In commercial dairies, especially in large-scale farms (sometimes over 3000 lactating cows), housing and management conditions are different from experimental farms that might influence cardiac activity in dairy cows in intensive housing systems. Buck et al. [10] studied the effects of the manure scraper on HRV. Their study was performed on experimental and commercial farms in field conditions.

In the present study, we aimed to assess the associations between two groups of variables: the first consisted of dependent variables of main interest (HRV parameters); the second has composed of explanatory variables that are supposed to influence the variables in the first group. We have chosen the studied factors on the basis of our earlier study [11] which concluded that the main factors affecting the welfare of the Hungarian dairy cows are feeding problems, lameness and cow comfort. The studied housing-related factors were group size, space allowance, milking frequency and season, whereas individual-related production factors included parity, daily milk yield, body condition score, locomotion score, and the physical activity of the animals. Our main objective was to detect how the mentioned explanatory variables associate with HRV indices, which would be helpful in the design of future experiments. Multivariate methods, such as canonical correlation analysis [12] cannot achieve this objective. However, formal methods to achieve this objective are available [13,14].

One such method is canonical correspondence analysis (CCOA) [15]. This is a generalization of the principal component analysis (PCA) with instrumental variables (e.g., explained variables) for counts or proportions, as proposed by Rao [13]. CCOA combines the ideas of multiple regression, MANOVA and PCA. It looks for linear combinations of the explanatory variables, which efficiently predict the matrix of outcome variables while simultaneously reducing that matrix [16]. CCOA is thus an example of direct gradient analysis, where the gradient in the studied variables is known a priori and the abundances of the dependent variables are considered to be a response to this gradient. To our knowledge, CCOA has been applied as a common statistical method in ecological research to study species-environment relationships [17–19] and in human epidemiology as well [20].

In this paper, we used CCOA to investigate the variability of housing- and individual-related factors mentioned above and to examine the associations between these and the most informative HRV indices describing the activity of the ANS in dairy cattle.

## Materials and Methods

### Farms and animals

The experiment was approved by the Department of Epidemiology and Animal Protection of the Directorate of Food Chain Safety and Animal Health at Central Agricultural Office (Permit Number: 22.1/1266/3/2010). All procedures involving animals were approved by the Ethics Committee of the Faculty of Veterinary Science, Szent István University. Measurements were carried out on five commercial dairy farms in Hungary on multiparous Holstein–Friesian cows between March 2011 and May 2013. Two farms were of smaller-scale with medium production (Farm 1 with 75 cows and Farm 2 with 80 cows) and three were larger-scale intensive production farms (Farm 3 with 850 cows, Farm 4 with 1900 cows and Farm 5 with 1200 cows).

Focal animals were selected from multiparous, clinically healthy, lactating animals for similar age, parity and days in milk (n = 50 for Farm 1, n = 40 for Farm 2, n = 65 for Farm 3, n = 60 for Farm 4, n = 45 for Farm 5, respectively). On Farm 1, cows were kept on pasture, the other farms had freestall barns. Cows were fed with a total mixed ration twice a day on all farms and water was available ad libitum. On all farms conventional milking systems were in operation during the study periods (Farms 1, 2 and 3: herringbone milking parlor, Farm 4: rotary milking parlor, Farm 5: parallel milking parlor).

Housing-related environmental factors and animal-related production factors are presented in Table 1. Space allowance was calculated for the whole barn (pasture) area, including the feeding place. Daily milk yield was recorded at the time of milkings. Locomotion of focal animals was scored on a 5-point scale according to Sprecher et al. [21] at the time the animals were exiting the milking parlor after the evening milking. The last author performed the scoring, who was trained to use this locomotion scoring system with on-farm experience. Body condition was scored at the time of fixing the heart rate monitors using the 5-point USA scoring system [22]. We visited both smaller and larger scale farms in each season (Farm 1: Aug–Nov; Farm 2: March–May, Farm 3: Oct–Dec, March–May; Farm 4: Oct–Nov, Farm 5: Apr–May). The number of studied animals per farm was equal in each season. We used the same protocol on every farm.

**Table 1:** Qualitative variables and housing- and animal-related factors (quantitative variables) involved in the analysis.

Qualitative variables (nominal factors)					
Physical activity: lying, lying + rumination, standing, standing + rumination, feeding					
Season: spring, summer, autumn, winter					
Farm No.	Farm 1	Farm 2	Farm 3	Farm 4	Farm 5
Quantitative variables					
Housing-related variables					
Group size	75	37	125	240	140
Space allowance (m^2^ per cow)	55.0	14.6	9.7	6.5	9.2
Milking frequency per day	2	2	2	3	3
Individual-related variables					
Parity	2.7±1.1	2.4±0.6	2.3±0.5	2.6±0.4	2.4±0.7
Daily milk yield (kg)	24.5±4.2	23.6±4.4	31.1±6.1	42.0±6.8	33.5±4.8
Body condition score^a^	3.0±0.3	2.5±0.6	2.6±0.4	3.1±0.4	2.7±0.3
Locomotion score^b^	2.6±0.5	2.8±0.9	2.4±0.5	3.0±0.4	2.7±0.3

^a^ ranging from 1 = very lean to 5 = fat

^b^ ranging from 1 = healthy to 5 = severely lame

### Monitoring of cardiac activity

IBIs were recorded with the Polar Equine RS800 CX heart rate receiver and a Polar Equine T56H transmitter (Polar Electro Oy, Kempele, Finland), with two integrated electrodes and a specific transmitter. Heart rate monitors were fitted to cows in individual insemination stalls after the morning milking. After soaking the body surface under the electrodes with tap water, electrode sites were covered with ultrasound transmission gel (Aquaultra Blue, MedGel Medical, Barcelona, Spain) without shaving the skin as advised by earlier reviews [4,5]. The positive electrode was located at the right scapula and the negative electrode at the cardiac area. The electrode belt was then fixed with a strong leather girth, which also contained a pocket for the heart rate receiver. The animals were then released back to their own production group. They were identified by numbers on their hind legs and backs drawn on at the time of fixing the heart rate monitors.

After moving cows back to the barns or the pasture, any kind of disturbance or any unnecessary contact with animals throughout the data collection period was avoided. IBI signals from the first two hours after fixing the heart rate monitors were excluded from later analysis, due to the influential effect that human interaction and the exploratory behavior of group mates towards the girth and device as novelties might have had on heart rate [23]. The IBI recording has finished when the belt was removed from the cows having returned from the evening milking.

### Behavioral observations

Behavioral observations were done by four experimenters at a time (two animals for each observer), Posture and activity of the cows were recorded throughout the whole length of HR recordings. Observers stood at least 6 m from the cows. They used watches, which were synchronized with the HR receivers to register the exact starting and end points of the animal’s actual behavior (posture/activity). Observers also recorded the time point of any disturbances occurring (e.g. a stockperson walked close by an experimental cow, interactions with group mates either as a performer or a receiver). To define the interactions with group mates (head butting, chasing, displacement, etc) the definitions of the Welfare Quality protocol were used [24]. According to level of physical activity, five categories were distinguished in the HRV analysis: lying, lying and rumination, standing, standing and rumination and feeding. For the categories ‘lying’ and ‘standing’ the following two criteria were established: 1) the cow is lying/standing comfortably without any disturbance from herd mates; 2) the cow has finished feeding and/or walking at least 5 min before the start of data recording. In the last 2 min before the periods of interest and throughout the periods no environmental effects or disturbances are observed.

### Processing of IBI data

The segments of HR recordings matching the periods of uninterrupted display of the studied posture/activity (undisturbed from group mates, stockmen, or sudden noise) were used for HRV analysis. A 2-min interval after any kind of disturbance or social interaction and a 5-minute interval after changing posture/activity were excluded from analysis. We examined equal lengths of 5 min periods as recommended for analysis of HRV [4,25]. Longer periods of undisturbed recording were subdivided into several 5-min segments. The circadian rhythm of cardiac activity [26] was taken into consideration when selecting valid IBI samples for HRV analysis. Four segments were chosen for every level of physiological activity per animal on each farm. Two IBI samples were analyzed for morning (between 9 a.m. and 1 p.m.) and two for afternoon (between 1 and 5 p.m.) periods (a total of 5200 samples).

The Kubios HRV software (version 2.2, Biomedical Signal Analysis Group, Department of Applied Physics, University of Kuopio, Finland) was used for HRV analysis [27]. Ectopic heartbeats and artifacts were corrected as described earlier [28]. Besides time domain measures [HR and vagal tone parameter root mean square of successive differences (RMSSD) between the successive IBIs] frequency-domain indices of HRV were calculated. IBI data were subjected to Fast Fourier Transformation of power spectrum analysis [29]. Spectral parameters included the normalized power of the high-frequency band (HF) for detecting tendencies in vagal activity [4–6] and the relative power of the low frequency (LF) component and HF (LF/HF ratio), which is indicative for the sympathovagal balance in dairy cattle [5]. Recommendations of von Borell et al. [4] were considered by setting the limits of the spectral components as follows: LF: 0.05–0.20 Hz and HF: 0.20–0.58 Hz. For graphical representation of the correlation between successive IBIs, where each interval in the time series (IBIi) is plotted against its successor (IBIi+1) the standard deviation 1 (SD1) was calculated by Poincaré plot analysis as described in earlier reviews on humans [25] and on farm animals [4]. SD1 also represents vagal nerve activity [27] and has been used in dairy cows [30–32].

### Statistical evaluation

All analysis were performed in the PAST Software Package [33,34]. For the evaluation of the effects of quantitative and qualitative factors on the animal’s cardiac activity the CCOA method was chosen [35]. The explanatory data set included ten variables and was subdivided into four submatrices (Table 1). The interpretation of relationships between explanatory variables and the five ANS-related HRV parameters is based on the correlation between the levels of explanatory variables and the CCOA factors [16]. The contribution of each explanatory variable to the explained variation in the HRV parameter data was determined by the inertia from marginal and conditional effects. Marginal effects are the amount of variation the variable explains singly. Conditional effects show the amount of additional variation each variable contributes when it is added to the model [36]. Season, farm and levels of physical activity as qualitative (nominal) variables were projected on the ordination space of HRV parameters. Results of CCOA are presented in an ordination diagram (triplot) where the ordination axes (Axes 1, 2, 3 and 4) are linear combinations of the explanatory variables. For testing the goodness of the ordination axes the significance level was set to 0.05.

## Results

### Explained variance of ordination axes and distribution of objects

The canonical eigenvalues and the explained variance for the 4 axes are presented in Table 2. Although Axis 3 was significant, the 94.28% of the total variance was explained by the first two axes, therefore we decided to use only Axes 1 and 2 as principal axes and displayed in the ordination diagrams hereinafter. Axis 3 does not provide other or substantially new conclusional possibilities.

**Table 2:** Eigenvalues and explained variance by the ordination axes.

Axis	Canonical eigenvalue	%	P-value
1	0.010926	81.745	0.0099
2	0.001673	12.525	0.0099
3	0.000766	5.734	0.0099
4	0.000023	0.001	0.2178

**Fig 1:**
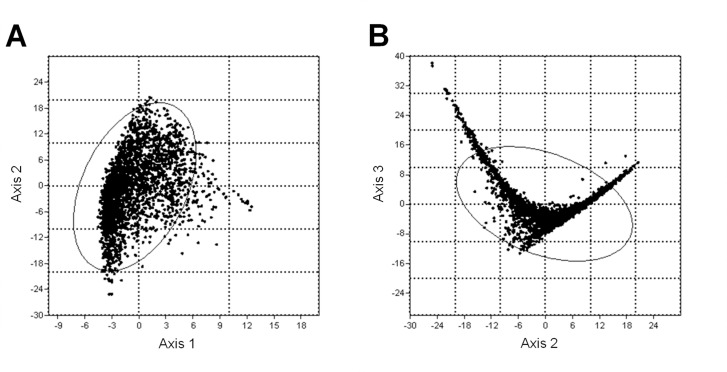
The ordination of objects for the dimension of axes 1–2 (A) and axes 2–3 (B). Concentration ellipses enclose 95% of the points.

From the CCOA analysis, associations between explanatory variables and HRV parameters were identified and presented in ordination diagrams. The ordination of objects both for the dimension of axes 1–2 and axes 2–3 appeared to be non-clustered (Fig 1), therefore the structure of the gradient-space in which each object has given values for one or more (housing- or individual-related) variables was organized homogenously in our study.

### Ordination of explanatory and dependent variables

**Fig 2:**
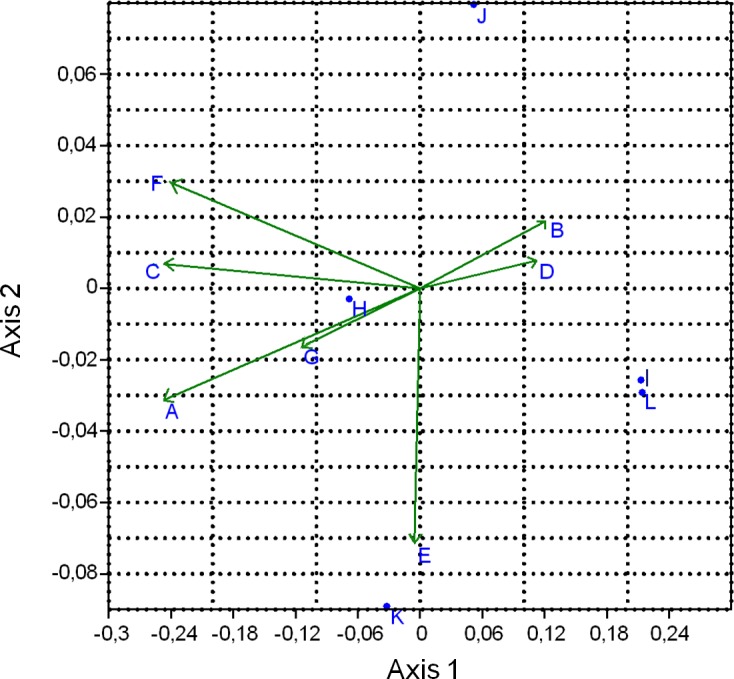
Canonical Correspondence Analysis (CCOA) ordination diagram showing the first two axes of the CCOA. Axis 1 and Axis 2 are linear combinations of the explanatory variables (A: group size, B: space allowance, C: milking frequency, D: locomotion score, E: body condition score, F: daily milk yield, G: parity). Dependent variables: H: HR, I: RMSSD, J: HF, K: LF/HF, L: SD1). The explained variance of the principal axes [Axis 1 (horizontally) and Axis 2 (vertically)] are 81.75% and 12.53%, respectively; for Axis 3 (not displayed) is 5.73%. The length of the vectors indicate the strength of the correlation between the quantitative environmental variables and the CCOA axes.

The ordination of explanatory variables and HRV indices to the first principal axes is presented in Fig 2. To Axis 1 (explained variance: 81.74%) high values of HR and low values of RMSSD and SD1 are accompanied by high values of group size, parity, milking frequency and milk yield parallel with the low values of space allowance and locomotion score. To Axis 2 (explained variance: 12.52%) high values of LF/HF and low values of HF are associated with high values of body condition score.

### Mapping of explanatory variables in the space of HRV parameters

**Fig 3:**
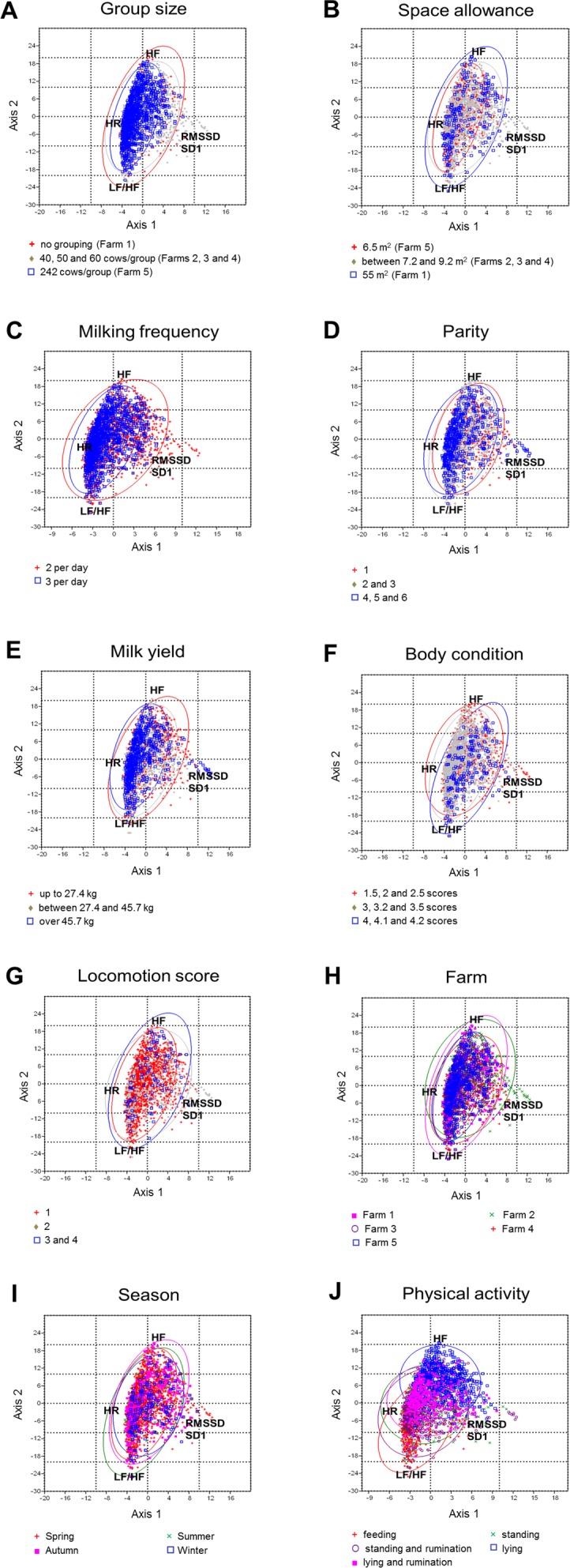
Graphical representation of the associations between the explanatory variables and HRV parameters using the first two axes obtained from CCOA. A, Group size; B, Space allowance; C, Milking frequency; D, Parity; E, Milk yield, F, Body condition; G, Locomotion score; H, Farm; I, Season; J, Physical activity. The ordination axes are linear combinations of the explanatory variables. In case of quantitative variables red + indicates values over the upper quartile (25% of objects), grey rhomb indicates the values between the upper and lower quartile (median, 50% of objects), and blue square indicates values lower than the lower quartile (25% of objects). Season, farm, and levels of physical activity as nominal variables were projected on the ordination space of HRV parameters. On each triplot concentration ellipses are displayed which enclose 95% of the points, assuming bivariate normal distribution of each subcloud.

The ordination of the housing- and individual-related variables in the spaces of cardiac parameters is shown in Fig 3.

The most prominent associations with HRV parameters were shown by the levels of physical activity of the cows (Fig 3J). Objects ordinate from the high values to low values of HR and LF/HF in the following order: feeding, standing and rumination, standing, lying and ruminating, lying. From the low values of SD1, RMSSD and HF to high values the same pattern can be observed. Seasons showed a similar but less pronounced tendency in the order of summer–autumn–spring–winter, and winter showed the lowest degree of variance from all seasons (Fig 3I).

When focusing on Farms, the greatest differences can be observed between Farms 1 and 4 (Fig 3H): Farm 1 is characterized by higher values of objects of vagus-related time-domain parameters (RMSSD, SD1), while Farm 4 is characterized by higher values of the objects of HR and LF/HF.

Higher values of parity are associated with higher sympathetic tone (higher values of HR, LF/HF, Fig 3D). Lower values of group size (especially on Farm 1, where animals were on pasture, without grouping, Fig 3A) and milking frequency (Fig 3C) showed lower degree of variance (positioned less concentrated) in the space of HRV parameters than objects representing higher values of these explanatory variables. Objects representing higher values of space allowance (Fig 3B) and locomotion scores (Fig 3G) have a larger variance than objects, which are characterized with lower values. Body condition was not associated with any of the HRV measures (Fig 3F).

## Discussion

In this work, we evaluated the associations between housing- and individual-related observational data (explanatory variables) and several HRV indices (response or dependent variables) of potential importance in cow welfare by reflecting on ANS activity in dairy cows from five commercial farms. To analyze the two groups of variables, one being explained by the second in a correspondence analysis framework, the CCOA method was chosen, which is extensively applied in plant [37,38] and animal ecology [39,40]. The CCOA method that we have used is a generalization of redundancy analysis [41] and have been used in human epidemiology [42]. As the first two principal axes explained more than 94% of total variance of the objects, we decided to use only those when analyzing the associations between the explanatory and cardiac variables. CCOA is only rarely used in animal science [43], and since our paper is the first that describe HRV using CCOA in animals, we have compared the associations with previous studies on HRV, using other analytical methods.

In our study, objects ordinate for both the dimension of axes 1–2 and axes 2–3 in a non-clustered manner, therefore the structure of the investigated variables was homogenous. Earlier studies reported that rare objects are often positioned as outliers in the CCOA ordinations, which gives the impression that they are highly influential [44], but in our case none of the objects was found to be outside the gradient-space contributing to unbiased results. In accordance with previous findings, it seems that *physical activity* involved in standing [9]–and in the present work in feeding and rumination as well–is associated with HRV parameters and HR emphasizing the importance of physical activity on cardiac function, which has to be considered in further cattle studies investigating stress. Similarly to physical activity, HRV was associated with *season*. Objects representing summer ordinate to high sympathetic activity (positioned close to HR and LF/HF in the CCOA triplot) and to low vagal activity (positioned far from SD1, RMSSD and HF). Objects representing autumn, spring and winter tended to be associated with higher vagal activity (positioned close to SD1, RMSSD and HF), following this order, respectively (Fig 3I).

Regarding *individual-related variables*, higher HR and lower values of RMSSD and SD1 were associated with higher values of parity and milk yield, and low values of locomotion score (Fig 2). Individuals with higher parity (Fig 3D) and milk yield (Fig 3E) and lower locomotion score (lower degree of lameness, Fig 3G) were characterized by a higher sympathetic and a lower vagal tone. Contrary to our results, other studies did not find correlation between HRV parameters and milk yield or stage of lactation [6] or the parity of the cows [30]. The small sample size (6–6 animals/experimental groups) and the high individual variability of HRV indices might be a reason for their findings. Similarly to our previous results [45], body condition was not associated with any of the HRV measures in our recent study. Body condition score–to our best knowledge–has not been measured in any HRV research performed in dairy cattle. Hagen et al. [6] found that body weight as a covariate had significant effect on HRV parameters, though body weight is different from body condition score.

In our recent study on lameness-related changes in HRV, we observed lower vagal tone in non-lame animals compared to lame ones [45]. The distribution of objects representing individuals with higher locomotion scores were more heterogenic in the space of HRV indices, therefore we can conclude, that lame animals are characterized with more variable cardiac function regarding the studied parameters and therefore (and also because of their higher stress vulnerability) the involvement of these animals is not recommended in cattle HRV studies.

When focusing only on the ordination of objects to Axis 1 (explained variance: 81.74%) time-domain measures of HRV were in association with most of the *housing-related explanatory variables*. Higher heart rates and lower values of RMSSD and SD1 were associated with high values of group size, milking frequency and low values of space allowance (Fig 2). These results indicate a higher sympathetic and a lower vagal tone of animals kept in larger groups, with lower space allowance and being milked three times a day than in animals housed in smaller groups with higher space allowance and milked two times a day. Farm 1 and Farm 4 were at two extremes of space allowance and group size, yet, we have to state that other unrecorded factors may also have systematically differed between farms. For group sizes greater than 100 cows, stress may arise from the failure to establish a stable dominance hierarchy [46] due to the unability to recognize all group mates. Regrouping of the animals may be frequent in an intensively managed large dairy herd, with large groups. Behavior of the cows is altered after regrouping [47], which may also be a source of stress and result in elevated sympathetic tone. These factors might occur more frequently on larger farms; however, neither social behavior nor the presence or frequencies of regroupings were recorded in our study. According to the work of Grant and Albright [46], the optimal size of a group of cows on any dairy, from a behavioral perspective, will be among others a function of 1) competition for space in the barn or pasture, 2) competition for feed and water, 3) availability of comfortable, usable freestalls. On the farms in our study, the TMR was provided two times a day, except for farm 4, where after the morning delivery, the TMR was pushed up every 30 minutes by an automatic robot. This system may promote numerous smaller meals, and therefore higher activity throughout the day [46], that may have effect on HRV patterns, however the feeding times and the number of meals/day were not measured.

According the study of Telezhenko et al. [48] the animals kept in larger pens move more and greater distances, regardless to the group size (number of the animals within the group). The space allowance and also the pen size was much greater on farm 1 compared to the other extreme, farm 4, though on all farms the group size were adjusted to the number of available resting places (freestalls, or the area of straw bedded yard), bunk space or the number of drinkers. Rushen et al. [49] showed in their study that aversive handling of dairy cows increased the fear of humans. On larger farms, one stockperson is responsible for and has impact on higher number of animals, possibly affecting their welfare [50]. In their study, Breuer et al. [51] showed that fear of humans accounted for 19% of the variation in milk yield between farms. Also, interaction between human and cattle are different if they happen outdoors than inside the barn, as their body looks smaller to animals outdoors, and the effect of human hands, voice and smell is less remarkable outdoors than inside (Seabrook and Mount, 1993, cited by [50]). In this study, the human-animal reactions were not measured, since we wanted to find associations between some easily recordable variables (such as group size and space allowance) and the HRV indices. Though the statistical analysis describes a similarity in patterns, not causal relations between variables, it is a limitation of the study that above mentioned differences that likely existed between farms were not taken into account.

We found, that objects representing lower values of group size and milking frequency and higher values of space allowance showed higher variance in the space of HRV parameters compared to those associated with higher values of group size and milking frequency and lower values of space allowance. This suggests that in case of larger number of cows in groups and higher number of milkings per day can lead to more homogenous values of HRV parameters. Therefore, these housing-related variables are recommended to be considered for reducing the variance of HRV data, especially, when smaller number of animals could be involved. Due to the limited number of housing related variables considered and the low number of farms involved, we can treat our results as promising conclusions of a pilot study, that draw the attention to the importance of farm-related variables in the HRV studies, and that should be verified in further experiments. Based on all of the above, we suggest to consider the following variables in further HRV studies on dairy cattle:

1. Great importance: physical activity, space allowance, group size, milking frequency, parity milk yield, locomotion score;
2. Moderate importance: season;
3. Low importance: body condition score, farm.

As all of the investigated factors could easily be registered on-farm, our results might be valuable for further field studies on dairy cattle HRV carried out on commercial dairies. Our findings indicate that the CCOA method is useful in demonstrating trends to guide more specific field studies on cattle well-being.

## References

[1] Porges SW. The polyvagal theory: phylogenetic contributions to social behavior. Physiol Behav. 2003;79: 503–513. 12954445 10.1016/s0031-9384(03)00156-2

[2] Kleiger RE, Stein PK, Bigger JT Jr. Heart Rate Variability: Measurement and Clinical Utility. Ann Noninvasive Electrocardiol. 2005;10: 88–101. 15649244 10.1111/j.1542-474X.2005.10101.x PMC6932537

[3] Koolhaas JM, Bartolomucci A, Buwalda B, De Boer SF, Flügge G, Korte SM, et al Stress revisited: a critical evaluation of the stress concept. Neurosci Biobehav Rev. 2011;35: 1291–1301. 10.1016/j.neubiorev.2011.02.003 21316391

[4] Borell von E, Langbein J, Després G, Hansen S, Leterrier C, Marchant-Forde J, et al Heart rate variability as a measure of autonomic regulation of cardiac activity for assessing stress and welfare in farm animals: a review. Physiol Behav. 2007;92: 293–316. 17320122 10.1016/j.physbeh.2007.01.007

[5] Kovács L, Jurkovich V, Bakony M, Póti P, Szenci O, Tőzsér J. Welfare assessment in dairy cattle by heart rate and heart rate variability–Literature review and implications for future research. Animal. 2014;8: 316–330. 10.1017/S1751731113002140 24308850

[6] Hagen K, Langbein J, Schmied C, Lexer D, Waiblinger S. Heart rate variability in dairy cows–influences of breed and milking system. Physiol Behav. 2005;85: 195–204. 15894344 10.1016/j.physbeh.2005.03.019

[7] Mialon MM, Deiss V, Andanson S, Anglard F, Doreau M, Veissier I. An assessment of the impact of rumenocentesis on pain and stress in cattle and the effect of local anaesthesia. Vet J. 2012;194: 55–59. 10.1016/j.tvjl.2012.02.019 22513300

[8] Sutherland MA, Rogers AR, Verkerk GA. The effect of temperament and responsiveness towards humans on the behavior, physiology and milk production of multiparous dairy cows in a familiar and novel milking environment. Physiol Behav. 2012;107: 329–337. 10.1016/j.physbeh.2012.07.013 22939763

[9] Frondelius L, Järvenranta K, Koponen T, Mononen J. The effects of body posture and temperament on heart rate variability in dairy cows. Physiol Behav. 2015;139: 437–441. 10.1016/j.physbeh.2014.12.002 25481355

[10] Buck M, Friedl K, Steiner B, Gygax L, Weschler B, Steiner A. Influence of manure scrapers on dairy cows in cubicle housing systems. Livest Sci. 2013;158: 129–137.

[11] Jurkovich V, Fóris B, Végh Á, Könyves L, Brydl E. Assessing welfare quality in Hungarian dairy cattle herds. Magyar Állatorvosok Lapja. 2012;134: 605–613. (in Hungarian)

[12] Mardia KV, Kent JT, Bibby JM. Multivariate Analysis. London: Academic Press; 1979.

[13] Rao CR. The use and interpretation of principal component analysis in applied research. Sankhya Ser. 1964;A26: 329–359.

[14] Robert P, Escoufier Y. A unifying tool for linear-mulivariate statistical methods: the RV- coefficient. Appl Statist. 1976;25: 257–265.

[15] Ter Braak CJJ. Canonical correspondence analysis: a new eigenvector technique for multivariate direct gradient analysis. Ecology. 1986;67: 1167–1179.

[16] Lebreton JD, Sabatier R, Banco G, Bacou AM. Principal components and correspondence analyses with respect to instrumental variables: an overview of their role in studies of structure-activity and species-environment relationships In: Devillers J, Karcher W. editors. Applied Multivariate Analysis in SAR and Environmental Studies. Brussels: EEC; 1991, p. 85–114.

[17] Bernard A, Magnan P, Plante M, Bernatchez L. Canonical correspondence analysis for estimating spatial and environmental effects on microsatellite gene diversity in brook charr (Salvelinus fontinalis). Mol Ecol. 1999;8: 1043–1053.

[18] Moctezuma C, Hammerbacher A, Heil M, Gershenzon J, Méndez-Alonzo R, Oyama K. Specific Polyphenols and Tannins are Associated with Defense Against Insect Herbivores in the Tropical Oak Quercus oleoides. J Chem Ecol. 2014;40: 458–467. 10.1007/s10886-014-0431-3 24809533

[19] Pattiyage IA, Wai WH. Sampling at mesoscale physical habitats to explain headwater stream water quality variations: Its comparison to equal-spaced sampling under seasonal and rainfall aided flushing states. J Hydrol. 2014;519: 3615–3633.

[20] Sourial N, Wolfson C. Zhu B, Quail J, Fletcher J, Karunananthan S, Bandeen-Roche K, Béland F, Bergman H. Correspondence analysis is a useful tool to uncover the relationships among categorical variables. J Clin Epidemiol. 2010;63: 638–646. 10.1016/j.jclinepi.2009.08.008 19896800 PMC3718710

[21] Sprecher DJ, Hostetler DE, Kanneene JB. A lameness scoring system that uses posture and gait to predict dairy cattle reproductive performance. Theriogenology. 1997;47: 1179–1187. 16728067 10.1016/s0093-691x(97)00098-8

[22] Hady PJ, Domecq JJ Kaneene JB. Frequency and precision of body condition scoring in dairy cattle. J Dairy Sci. 1994;77: 1543–1547. 8083413 10.3168/jds.S0022-0302(94)77095-8

[23] Mohr E, Langbein J, Nürnberg G. Heart rate variability: A noninvasive approach to measure stress in calves and cows. Physiol Behav. 2002;75: 251–259. 11890975 10.1016/s0031-9384(01)00651-5

[24] Welfare Quality: Assessment protocol for cattle Welfare Qualty Consortium. Lelystad, The Netherlands, 2009.

[25] Task Force of the European Society of Cardiology, North American Society of Pacing and Electrophysiology. Heart rate variability: standards of measurement, physiological interpretation, and clinical use. Circulation. 1996;93: 1043–1065. 8598068

[26] Piccione G, Grasso F, Giudice E. Circadian rhythm in the cardiovascular system of domestic animals. Res Vet Sci. 2005;79: 155–160. 15924933 10.1016/j.rvsc.2004.11.010

[27] Tarvainen MP, Niskanen J-P, Lipponen JA, Ranta-aho PO, Karjalainen PA. Kubios HRV–Heart rate variability analysis software. Comp Meth Prog Biomed. 2014;113: 210–220. 10.1016/j.cmpb.2013.07.024 24054542

[28] Kovács L, Tőzsér J, Szenci O, Póti P, Kézér FL, Ruff F, et al Cardiac responses to palpation per rectum in lactating and nonlactating dairy cows. J Dairy Sci. 2014;97: 6955–6963. 10.3168/jds.2014-8327 25200771

[29] Akselrod S, Gordon D, Ubel FA, Shannon DC, Berger AC, Cohen RJ. Power spectrum analysis of heart rate fluctuation: a quantitative probe of beat-to-beat cardiovascular control. Science. 1981;213: 220–222. 6166045 10.1126/science.6166045

[30] Minero M, Canali E, Ferrante V, Carenzi C. Measurement and time domain analysis of heart rate variability in dairy cattle. Vet Rec. 2001;149: 772–774. 11808666

[31] Kovács L, Bakony M, Tőzsér J, Jurkovich V. Short communication: The effect of milking in a parallel milking parlor with non-voluntary exit on the HRV of dairy cows. J Dairy Sci. 2013;96: 7743–7747. 10.3168/jds.2013-7030 24140325

[32] Kovács L, Tőzsér J, Kézér FL, Ruff F, Aubin-Wodala M, Albert E, et al Heart rate and heart rate variability in multiparous dairy cows with unassisted calvings in the periparturient period. Physiol Behav. 2015;139: 281–289. 10.1016/j.physbeh.2014.11.039 25449409

[33] Hammer Ø, Harper DAT. Paleontological Data Analysis. Oxford: Blackwell Publishing; 2006.

[34] Hammer Ø, Harper DAT, Ryan PD. PAST: Paleontological Statistics Software Package for Education and Data Analysis. Palaeontol Electron 2001;4:9.

[35] Legendre P, Legendre L. Numerical Ecology. 2nd edition Amsterdam: Elsevier; 1998.

[36] ter Braak CJF, Smilauer P. CANOCO 4. Wageningen: Centre for Biometry; 1998.

[37] Cooper A, McCann T, Ballard E. The effects of livestock grazing and recreation on Irish machair grassland vegetation. Plant Ecol. 2005;181: 255–267.

[38] Marler TE, Del Moral R. Primary succession in Mount Pinatubo: Habitat availability and ordination analysis. Commun Integr Biol. 2013,6:e25924 10.4161/cib.25924 24505499 PMC3913662

[39] D’Ambrosio JL, Williams LR, Williams MG, Witter JD, Ward AD. Geomorphology, habitat, and spatial location influences on fish and macroinvertebrate communities in modified channels of an agriculturally-dominated watershed in Ohio, USA. Ecol Eng. 2014;68: 32–46.

[40] Lunardi VO, Macedo RH, Granadeiro JP, Palmeirim JM. Migratory flows and foraging habitat selection by shorebirds along the northeastern coast of Brazil: The case of Baía de Todos os Santos. Estuar Coast Shelf Sci. 2012;96: 179–187.

[41] Van der Wollenberg AL. Redundancy analysis. An alternative for canonical correlation analysis. Psychometrika. 1977;42: 207–219.

[42] Friedman BH, Thayer JF. Facial muscle activity and EEG recordings: redundancy analysis. EEG Clin Neurophysiol. 1999;79: 358–360. 10.1016/0013-4694(91)90200-n 1718708

[43] Faye B, Lescourret F, Dorr N, Tillard E, MacDermott B, McDermott J. Interrelationships between herd management practices and udder health status using canonical correspondence analysis. Prev Vet Med. 1997;32: 171–192. 9443326 10.1016/s0167-5877(97)00017-2

[44] Greenacre M: The contributions of rare objects in correspondence analysis. Ecology. 2013;94: 241–249. 23600258 10.1890/11-1730.1

[45] Kovács L, Kézér FL, Jurkovich V, Kulcsár-Huszenicza M, Tőzsér J. Heart Rate Variability as an Indicator of Chronic Stress Caused by Lameness in Dairy Cows. Plos One 2015;10(8): e0134792 10.1371/journal.pone.0134792 26270563 PMC4536120

[46] Grant RJ, Albright JL. Effect of animal grouping on feeding behavior and intake of dairy cattle. J Dairy Sci. 2001;84 (E. Suppl.): E156–E163.

[47] Keyserlingk MAG, Olenick D, Weary DM. Acute behavioral effects of regrouping dairy cows. J Dairy Sci. 2008; 91: 1011–1016. 10.3168/jds.2007-0532 18292257

[48] Telezhenko E, Keyserlingk MAG, Talebi A, Weary DM. Effect of pen size, group size and stocking density on activity in freestall-housed dairy cows. J Dairy Sci. 2012; 95: 3064–3069. 10.3168/jds.2011-4953 22612942

[49] Rushen J, Munksgaard L, de Pasillé AMB. Fear of people by cows and effects on milk yield behavior and heart rate at milking. Appl Anim Behav Sci. 1999; 82: 720–727. 10.3168/jds.S0022-0302(99)75289-6 10212458

[50] Raussi S. Human-cattle interactions in group housing. Appl Anim Behav Sci. 2003; 80: 245–262.

[51] Breuer K, Hemsworth PH, Barnett JL, Matthews LR, Colemann GJ. Behavioural response to humans and the productivity of commercial dairy cows. Appl Anim Behav Sci. 2000; 66: 273–288. 10700627 10.1016/s0168-1591(99)00097-0

